# A Practical Guide to Analyzing Time-Varying Associations between Physical Activity and Affect Using Multilevel Modeling

**DOI:** 10.1155/2018/8652034

**Published:** 2018-07-09

**Authors:** Jinhyuk Kim, David Marcusson-Clavertz, Fumiharu Togo, Hyuntae Park

**Affiliations:** ^1^Department of Biobehavioral Health, The Pennsylvania State University, University Park, PA, USA; ^2^Department of Psychology, Lund University, Lund, Sweden; ^3^Educational Physiology Laboratory, Graduate School of Education, The University of Tokyo, Tokyo, Japan; ^4^Department of Health Care and Science, College of Health Science, Dong-A University, Busan, Republic of Korea

## Abstract

There is growing interest in within-person associations of objectively measured physical and physiological variables with psychological states in daily life. Here we provide a practical guide with SAS code of multilevel modeling for analyzing physical activity data obtained by accelerometer and self-report data from intensive and repeated measures using ecological momentary assessments (EMA). We review previous applications of EMA in research and clinical settings and the analytical tools that are useful for EMA research. We exemplify the analyses of EMA data with cases on physical activity data and affect and discuss the future challenges in the field.

## 1. Introduction

Enabled by technological developments, ecological momentary assessment (EMA) [1] using mobile data collection has become an essential research tool in many fields of social and behavioral sciences and is continuing to spread to other areas of sciences. EMA research covers a wide range of phenomena, including the study of environmental, physical, physiological, psychological, and sociological factors, using repeated or continuous recording. Given the widespread use of EMA methods across the different sciences, various terms have been used to refer to similar procedures. EMA methods focusing on self-report data are frequently referred to as experience-sampling methods (ESM) [2], whereas those focusing on physical, physiological, or biological data are often called ambulatory assessments (AA) [3]. However, we use the term EMA broadly to include all these types of ecological, intensive assessments. To exemplify, EMA studies investigate various behaviors, experiences, and environmental conditions, including depression [4–6], psychological stress [7, 8], self-esteem [9], diet [10], self-reported physical activity [11, 12], smoking [13–15], sexual behavior [16], compulsive buying [17], social interaction [4, 18], work activity and satisfaction [8, 19], diabetes management [18, 20], effects of medication [21, 22], asthma [23, 24], allergies [25, 26], tinnitus [27], and working memory and attention [28]. In addition, technological developments have enabled automated EMA of behaviors (e.g., taking medication [29]) and physical environment (e.g., air sampling [26], sampling in electromagnetic fields [30]). Ambulatory monitoring of cardiovascular function, using portable cardiac monitors, has been used for several decades as a tool for understanding the association between experiences and cardiovascular health [31]. Recent developments have expanded physiological monitoring to other parameters, such as physical activity [32–38], hypothalamic-pituitary-adrenal axis activity [39–42], blood glucose [43], skin temperature [44], pulmonary function [23], and others. Furthermore, these data collections are widely used to evaluate treatment and intervention of crucial health-related behaviors in health psychology and behavioral medicine, such as coping with illness and treatment [45, 46], medication compliance [47, 48], and exercise [49, 50]. Psychiatric (or psychosomatic) disorders studied with EMA include a wide range of psychopathology, such as addictive disorders [51, 52], gastrointestinal disorders [53], sexual dysfunction [54], eating disorders [46, 55, 56], attention deficit hyperactivity disorder (ADHD) [57, 58], mood dysregulation [59], anxiety disorders [60–62], depressive disorders [63–65], bipolar disorder [66], and schizophrenia [67–69].

Why did EMA become frequently used in various areas of researches including clinical settings? One advantage of EMA methods is that they enable us to study a phenomenon in its natural environment. A second advantage is that they allow us to study the time course of target variables. Intensive data collection enables the exploration of development trajectories of psychiatric disorders and physical health conditions and identification of factors that are predictive of these trajectories. For instance, one study used EMA methods to examine if spousal responsiveness to verbal expressions of pain in patients with knee osteoarthritis predicted patients' physical function over time [70]. Such fluctuations in the trajectories cannot be captured by traditional, cross-sectional data collection methods. A third advantage is that EMA enable us to assess related symptoms with other related factors (e.g., physiological states or social and environmental situations) immediately before and just after disorders (e.g., panic attacks [62], binge eating [55]), which give us important insights into pathogenic processes and prevention of psychiatric disorders and poor physical health.

Many EMA studies have examined how a phenomenon covaries with variables that may vary across different levels, including moments (e.g., mood states), days (e.g., work days versus weekends), persons (e.g., unemployed versus employed), or other levels (e.g., organizations, seasons) in various populations, including patients with psychiatric disorders and physical conditions. For example, cardiovascular reactivity [71–74] and cortisol-related reactivity [39–42, 75] were reported to be associated with levels of psychological stress, and changes in pulmonary functions tested by a spirometer were associated with daily positive/negative affect, as well as the symptom of shortness of breath in asthma patients. Health-related behaviors, such as eating [76, 77], smoking [13, 14], and alcohol consumption [78, 79], exhibited associations with variation in physical symptoms and psychological states, e.g., craving, positive/negative affect, and anxiety. Furthermore, associations between physical activity measured by self-report and daily fluctuations in psychological states have been reported [11, 12]. These studies provide strong evidence that biological/physiological measures vary in time with momentary symptoms. Thus, the existence of such objective proxies for subjective symptoms indicates the possibility of the practical use of them for monitoring momentary symptoms in a continuous fashion (i.e., without the need for self-reports). There might also be advantages in simultaneously using self-reported subjective symptoms and objective measures to improve the explanation of health outcomes.

**Figure 1:**
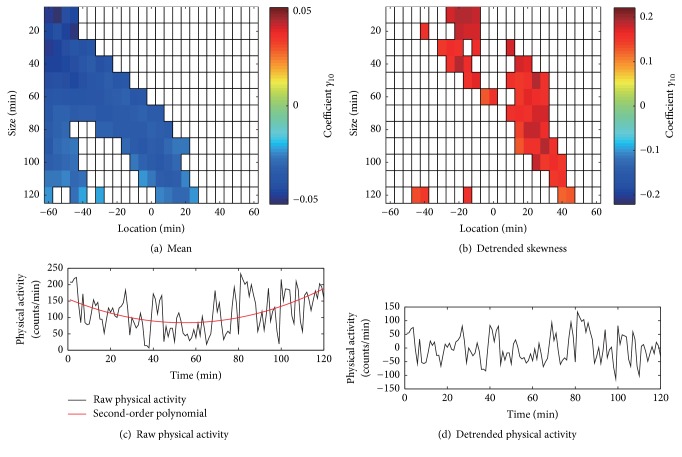
**Analytic techniques for physical activity.** (a) The temporal associations of depressive mood and local mean of physical activity which evaluates lower/higher mean activity levels. Estimated values of the univariate multilevel model coefficient for the associations are shown in a colored matrix form consisting of 25 columns (different location) and 12 rows (different size) in older adults (*n* = 9). Each grid cell indicates specific location and size of a time frame used for calculating the local mean of physical activity surrounding each EMA recording of depressive mood. A color in each cell represents the value of the model coefficient (*γ*_10_) of the predictors. The false discovery rate with the* q* value of .05 was used as the multiple comparison adjustment. Only the significant cases were shown by colors. Note that the univariate model used for the analysis is as follows. Depressive mood score_tj_ = *γ*_00_ + *γ*_10_ (local statistics of physical activity_tj_) + *ζ*_0j_ + *ε*_tj_ [see [85] for details]. (b) The same is shown in panels (a), except for the local mean. Local skewness of physical activity, which evaluates asymmetry of a distribution (i.e., occasional bursts of physical activity in a time window), was used in this panel. (c) A raw physical activity time series for 120 min and the second-order polynomial line (red). (d) The detrended physical activity derived by subtracting the fitted line for the original data.

It has been suggested that momentary fluctuations in behavioral data, specifically those on physical activity capturing bodily acceleration, reflect the dynamics of systems organizing human behavior and can be used to examine behavioral disorders, including mental illnesses [32–38]. Indeed, altered physical activity is one of the cardinal signs of psychiatric disorders and included in their diagnostic criteria [80]. For example, major depressive disorders (MDD) are characterized by the presence of symptoms associated with behavioral alterations, including diminished physical activity, psychomotor retardation or agitation, and sleep disturbances [80]. Specifically, several studies using accelerometer have been conducted with patients with depression, showing disruption of the circadian rhythm [32–34]. Research has shown the existence of robust statistical regularities concerning daily life behaviors, specifically how resting and active periods derived from physical activity data are interwoven into daily life [81]. In addition, this research found, in patients with MDD, a significant alteration of a parameter of the robust law representing the distribution of resting periods; compared to healthy subject, these patients exhibited more intermittent behavioral patterns characterized by reduced mean activity levels associated with occasional bursts of physical activity counts [81, 82]. Furthermore, alterations of intermittent properties of physical activity have been reported in schizophrenia and bipolar disorder [83, 84]. Recent studies showed the psychobehavioral correlates of temporal diurnal fluctuations in momentary depressive mood and behavioral dynamics [85, 86]. The results in these studies suggested that an increased intermittency of physical activity (i.e., low mean level and occasional burst of physical activity) appeared concurrently with the worsening of depressive mood in healthy subjects across a wide range of populations (adolescents, undergraduates, and adult office workers) [85], as well as in patients with MDD [86]. Furthermore, the cross validation between healthy subjects and patients with MDD were confirmed, indicating that the same psychobehavioral correlates are shared by both groups [86]. A pilot study suggested that temporal variations in depressive mood are affected by underlying changes in physical activity in older adults. Reduced activity patterns preceded or occurred concurrently with the worsening of depressive mood rather than following (Figures 1(a) and 1(b)) [87]. These findings suggest that physical activity obtained by accelerometer is a useful measure for evaluating behavioral abnormalities associated with psychiatric disorders, and that its characterization is likely to provide an objective measure for these disorders. However, other studies have not found support for associations between some types of mental disorders or psychological states and physical activity. For example, a study reported nonsignificant bidirectional associations between mood (i.e., energetic arousal, valence, and calmness) and physical activity in inactive university students [88]. Another study showed that physical activity contributes to an improvement of positive affect, but not a reduction of negative affect in MDD [89].

In this paper, we describe analytic models that are useful for analyzing EMA data with cases on physical activity data and affect. We also offer Supplementary Materials (available here) with SAS code for how to handle physical activity data obtained by accelerometer and use multilevel modeling techniques on EMA data.

## 2. Analytic Tools and Techniques for Evaluating Time-Varying Associations between Physical Activity and Affect

### 2.1. Multilevel Modeling

**Figure 2:**
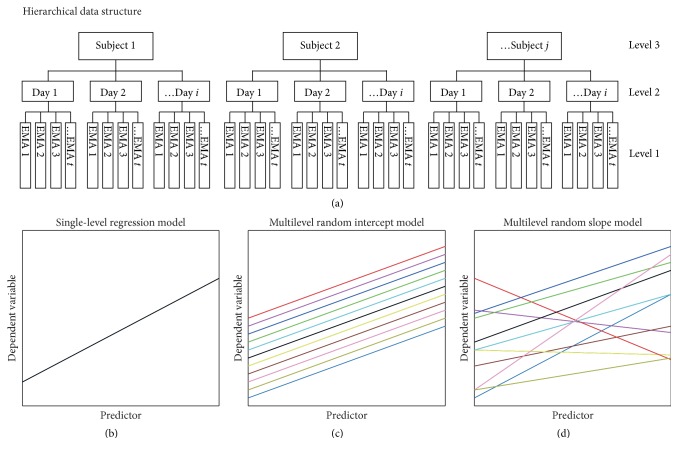
**Concept plots illustrate multilevel modeling using hierarchical ecological momentary assessment (EMA) data. **(a) An example of hierarchical data structure in which EMA observations (level 1) nested within days (level 2) nested within subjects (level 3). The number of EMA observations (*t*) and days (*i*) can be different in each subject. (b) Traditional regression model with fixed slope and intercept which do not vary across subjects. (c) Multilevel model with random intercepts, which vary across subjects, and fixed slopes. (d) Multilevel model with random intercepts and slopes. The multilevel model can be tested with random slopes and fixed intercepts, but the practical use of the model may be limited.

Although there are several analytical approaches to examine the association between physical activity and affect in daily life (e.g., correlation, regression, or time series modeling), multilevel modeling is suitable for addressing unbalanced and hierarchical EMA data. In EMA data, multiple observations are typically hierarchically nested within individuals, with the number and timing of observations varying between individuals (see Figure 2 for an example of EMA data structure). In addition, EMA studies usually have missing data due to difficulties in fully complying with the schedule. Traditional techniques such as repeated measures analysis of variance (RM-ANOVA) are not suitable for analyzing these unbalanced data sets [90]. However, such data can be handled by the multilevel modeling approach, which is an extension of traditional regression models and has been recommended for the analysis of data with a hierarchical structure (Figure 2) [90–92].

In multilevel modeling, these within- and between-individual effects can be handled together in the same model by incorporating random effects into model coefficients, i.e., allowing the coefficients to vary across individuals. For example, a researcher might expect that the average level of physical activity and the influence of negative affect on physical activity differ significantly between individuals, and therefore, model these effects as random intercepts and slopes, respectively. Although the multilevel model can be expressed as a single equation, it is easier to understand if it is initially presented as a set of equations separating within- and between-individual levels. In EMA analysis, usually observations are modeled as level 1 (within-individual level) units nested within individuals who are modeled as level 2 (between-individuals level) units. An example of multilevel models is as follows.

*Level 1 Equation (Within-Individual [Observation] Level)*

(1) $$\begin{array}{c} Y_{tj}=\pi_{0j}+\overset{n}{\underset{k=1}{\sum }}\pi_{kj}\left(\;X_{tj}^{k}-{\bar{X}}_{j}^{k}\;\right)+\varepsilon_{tj}\;\left(k=1,\ldots ,n\right) \end{array}$$

where* Y*_tj_ indicates the dependent variable (e.g., negative affect or depression)* t*th momentary observation for the* j*th subject; *X*_*tj*_^*k*^ is the* k*th predictor (e.g., physical activity;* k* represents the order of predictors) corresponding to the* t*th momentary observation for the* j*th subject; ${\overset{-}{X}}_{j}^{k}$ is the person mean of the* k*th predictor for centering to estimate the within-person effect of the predictor (physical activity) on the dependent variable of subjective symptoms [93];* n* is the total number of predictors; *π*_0j_ and *π*_k j_ are the subject* j'*s intercept and coefficient (i.e., slope) of the predictor, respectively; and *ε*_tj_ is the within-individual residual.

*Level 2 Equations (Between-Individual Level)*

(2) π0j=γ00+γ01Zj+ζ0j

(3) πkj=γk0+γk1Zj+ζkj

where *γ*_00_ is the average intercept across all subjects; *γ*_k0_ is the average slope across all subjects; *Z*_*j*_ is the between-individual level predictor representing, e.g., subject's characteristics; *γ*_01_ and *γ*_*k*1_ are the effect of the variable *Z*_*j*_; and the random terms *ζ*_0j_ and *ζ*_*kj*_ are the between-individual residuals.

*Combined Model*

(4) Ytj=γ00+γ01Zj+∑k=1nγk0Xtjk−X¯jk+∑k=1nγk1ZjXtjk−X¯jk+ζ0j+∑k=1nζkjXtjk−X¯jk+εtj

When the groups are nested within additional groups, the data form a 3-level hierarchy and 3-level models can be fitted to account for the additional level, e.g., EMA observations (level 1) nested within days (level 2) nested within individuals (level 3). An example of 3-level multilevel models is as follows (combined model is not shown).

*Level 1 Equation (Within-Individual [Observation] Level)*

(5) $$\begin{array}{c} Y_{tij}=\pi_{0ij}+\overset{n}{\underset{k=1}{\sum }}\pi_{kij}\left(\;X_{tij}^{k}-{\bar{X}}_{j}^{k}\;\right)+\varepsilon_{tij}\;\left(k=1,\ldots ,n\right) \end{array}$$

where *Y*_*tij*_ indicates the dependent variable at the *t*th momentary observation for the *j*th subject on the *i*th day; *X*_*tij*_^*k*^ is the *k*th predictor corresponding to the *t*th momentary observations for the *j*th subject on the *i*th day; *π*_0*ij*_ and *π*_*kij*_ are the subject *j*'s intercept and coefficient (i.e., slope) of the predictor on the *i*th day, respectively; and *ε*_*tij*_ is the observation-level residual.

*Level 2 Equations (Within-Individual [Day] Level)*

(6) π0ij=β00j+ζ0ij

(7) πkij=βk0j+ζkij

where *β*_00*j*_ is the subject *j*'s intercept. *β*_*k*0*j*_ is the subject *j*'s slope; and the random terms *ζ*_0*ij*_ and *ζ*_*kij*_ are the day-level residuals.

*Level 3 Equations (Between-Individual Level)*

(8) β00j=γ000+γ001Zj+δ00j

(9) βk0j=γk00+γk01Zj+δk0j

where *γ*_000_ and *γ*_k00_ are the average intercept and slope across all subjects, respectively;* Z*_j_ is the between-individual level predictor representing, e.g., subject's characteristics; *γ*_001_ and *γ*_k01_ are the effect of the variable* Z*_j_; and the random terms *δ*_00j_ and *δ*_k0j_ represent the residuals on the between-individual level. See SAS codes in the Supplementary Materials (available here) for the above models.

### 2.2. Which Statistics Should Be Used to Characterize Physical Activity?

Accelerometer is commonly used to objectively measure physical activity and capable of detecting large volumes of small changes in bodily acceleration. A common accelerometer method is to count zero-crossing activities; that is, the number of times that the signal crosses zero within the buffer [94], accumulated to 1-min epochs (we will assume this method in the discussion below, but there are also other ways to assess accelerometer data). The accelerometer enables opportunities to improve the characterization of activity patterns in daily life but also brings new analytic challenges despite expanding efforts to address these issues [95]. A study examined several issues with the use of accelerometer data on algorithms for the time of wearing or taking off the device and activity cut-off points for different intensities of physical activity [96]. The study showed that the choice of epoch length, which refers to the interval of time over which the units of accelerometer measures are aggregated (e.g., 15 seconds or 1 minute), may introduce significant errors when the chosen epoch length mismatches the length originally used for validating the wear time algorithm and activity cut-off points. This indicates that wear time or time spent in different intensities of physical activity cannot be directly compared across studies unless they used the same epoch lengths [96].

In addition to characterizing general activity patterns in daily life, accelerometers are useful tools for estimating the extent of a person's movement over a given period of time, including the intensity, duration, frequency, and the type of movement [95]. There has been growth in research on time spent in different intensities of physical activity (e.g., sedentary behavior [97–100], light, moderate, and vigorous physical activity [101–103]), but a common accelerometer measure is the activity counts per a certain period of time, which represent total volume of physical activity.

Although there are several important issues to consider when analyzing accelerometer data, we focus on how to characterize local (i.e., temporal) physical activity patterns surrounding EMA recordings of affect. To extract and characterize activity patterns in a temporal time window, researchers can analyze local statistics of physical activity data up to the fourth-order moment (i.e., mean, standard deviation [*SD*], skewness, and kurtosis) around EMA recordings (e.g., 60-min local mean of physical activity around the EMA signal). However, a research group focused on mean and skewness because they considered first- and third-order moments to be sufficient to characterize the observed accelerometer data [85, 86]. While* SD* (i.e., the second-order moment) is a standard measure characterizing variability of data, it can be inappropriate when the data do not approximate a normal distribution; the distribution of physical activity has nonnegative values, leading to a positively skewed distribution. Intermittency or non-Gaussianity in natural phenomena is known to be successfully captured by the higher-order statistics, such as nonzero skewness or larger kurtosis (flatness) of the probability distribution of the observed data [104, 105], corresponding to the presence of frequent bursts. Indeed, the local* SD* of physical activity did not play a major role in predicting affect (i.e., depressive mood) scores [85, 86]. In contrast, the skewness, as a measure of asymmetry of a distribution, is thought to be more appropriate to characterize the observed asymmetry. Lower or higher mean activity levels quantify the overall states of physical activity. Higher positive skewness quantifies occasional bursts of physical activity [81–83]. Other local statistics of physical activity that can capture the intermittency in physical activity more robustly, such as entropy-type nonlinear statistics, can also be considered.

It is important to consider the effect of time of day on physical activity. A simple way to address this would be by adding a term for time of day (e.g., every 4 hours or morning/afternoon/evening blocks) to (1) or (5) as a controller or moderator [106]. We can also use detrended activity data [85], where a diurnal trend of activity data is subtracted by fitting polynomial functions (e.g., the first-order polynomial to adjust a linear trend) before calculation (Figures 1(c) and 1(d)), which aims at eliminating effects of nonstationarity due to, e.g., daily activities; the effects up the higher-order polynomials can be systematically examined.

### 2.3. Considering Size and Location of Time Windows for Aggregation of Physical Activity Data

One of the most important questions when examining the association between two (or more) constructs varying over time is how to address the time windows (i.e., location and size) that are used to aggregate each construct. The choice of the size of the time window may be important because it could have a significant impact on the robustness of the statistics and their temporal coincidence with the symptoms. Time windows can be chosen either by using theoretical rationale or by explorative examination. An example of the latter is described below.

One possible attempt is to systematically vary the size and location of the time windows to examine their effects [85]. For example, when the epoch length of physical activity obtained by accelerometer is 1-min, 60-min local mean or* SD* of physical activity is computed from 60 data points, whereas 5-min local statistics are computed from 5 data points. Theoretically, the larger the size of the time window, the greater the stability and reliability of the estimates. However, the choice of larger time windows may obscure more transient fluctuations in the relations between physical activity and self-reported symptoms. Prior studies have used many different sizes of time windows to understand the associations between physical activity and affect states. There are many studies that have focused on very short time windows: 5-30 min [101, 103, 107, 108], which may be useful to check transient associations among target variables or examine health benefits from an even short period of physical activity. One study systematically varied the size of time windows from 5 min (transient) to 2 hours (medium) with a 5-min time interval to test proper time windows predicting depressive mood [85]. Another study used medium (4 hours) time windows of physical activity to compare with affect states assessed every 4 hours [11]. The associations between physical activity and affect on a day level (i.e., relatively long time window) have also been examined [109, 110]. Day-level time windows to aggregate physical activity may be used to examine overall associations with daily affect or event (e.g., sleep), but this examination is sometimes pragmatically made due to the limitation of sparse sampling (i.e., no observations within a day). The size of time windows largely depends upon the research question, but given the large freedom researchers typically have in selecting the size of time windows it is important that future research evaluates the reproducibility of the time-specific effects.

In addition, the choice of the location of the time window plays an important role in the investigation of causal associations, such as whether physical activity precedes or follows changes in momentary symptoms. There is scarcity of research on the bidirectional association between physical activity and momentary symptoms. Some studies showed that physical activity influences mental health benefits [108, 111], whereas others focused on how subjective symptoms predict subsequent physical activity [102, 108, 112]. However, it is a complicated domain and careful consideration of such a trade-off is important, although the optimal choice might be difficult to predict. Figures 1(a) and 1(b) are examples that show an examination of the temporal associations of depressive mood and local mean or detrended skewness of physical activity. Estimated values of the univariate multilevel model coefficient (i.e., slope) for the associations are shown in a colored matrix form consisting of 25 columns (different location) and 12 rows (different size). We considered 25 different locations (−60, −55, −50,…, 55, 60 min) and 12 different sizes (120, 110, 100,…, 20, 10 min). In total, we considered 300 combinations (25 locations × 12 sizes) for local statistics of physical activity to examine the association with depressive mood assessed by EMA. More specifically, the top left cell in Figure 1(a) represents the model coefficient for the association between depressive mood (EMA) and local mean which calculated from the 10-min size of time window 60 min before EMA (i.e., from −60 to −50 min before EMA). Thus, the colored matrices generally show reduced mean or (detrended) positively skewed activity patterns preceded or occurred concurrently with a higher level of depressive mood rather than following. The false discovery rate with the* q* value of .05 was used as the multiple comparison adjustment [113].

Although we discussed the size and location of time windows which are important when we explore the relationship with self-reported symptoms, the underlying mechanism of sustainability and causality alterations in the levels and patterns of physical activity with affect is uncertain. Further study using the data of high temporal resolution is necessary to clarify this question.

## 3. Further Challenges

Behavioral patterns characterized by reduced activity and intermittent bursts during low activity periods, as measured by accelerometer, are associated with EMA reports of worse depressive mood in healthy adolescents, older adults, undergraduates, office workers, and patients with MDD. This suggests that behavioral monitoring by the accelerometer may contribute to the identification of changes in subjective symptoms and improved management of these symptoms. While prior studies successfully provided a psychobehavioral measure based on accelerometer data, other types of time-varying changes in daily life should be examined to understand the associations between objective/subjective measures and health-related outcomes.

Many researchers and clinicians these days on the behavioral sciences and other scientific disciplines use mobile data collection incorporating information and communication technologies (ICTs), which enables a more refined understanding of psychiatric disorders including associations among various behavioral/physiological/biological measures. Furthermore, wearable devices (e.g., smartwatch) are increasingly popular to monitor health outcomes such as physical activity, sleep, and heart rate. The abundant information extracted from wearable devices is provided to numerous users often via smartphone applications and have great potential to elicit useful data for health outcomes in academic fields. Another challenge is how to use this information for improved monitoring, management, and intervention of health-related behaviors. For example, the concept of ecological momentary interventions (EMIs), in which real-time interventions are delivered to individuals during their everyday lives in natural settings, is a core elemental technology that is used for novel treatments of diseases including psychiatric disorders [114]. In addition, emerging electronic devices will make “context-sensitive prompting” possible, where questions are automatically triggered based on the subject's behavior, location, physiological states, past responses, and social interactions, which is considered useful for detecting early signs of psychiatric disorders and their pathological transitions [106, 115]. However, actual realization and examination for these novel techniques are necessary in further studies.

## 4. Conclusion

In this paper, we introduced the multilevel modeling approach, which is useful for analyzing EMA data with observations hierarchically nested within individuals. Although new analytic challenges arise with addressing accelerometer data, it allows for nuanced characterizing of the temporal pattern of physical activity and its correlates. We exemplified different kinds of statistics (e.g., mean and skewness) of physical activity to extract activity patterns in various temporal time windows (i.e., size and location around EMA) which can be widely used according to research questions, but further studies using different types of statistics with a high temporal resolution are necessary to clarify these issues. Detailed SAS codes of multilevel models are shown in the Supplementary Materials.

## Supplementary Materials

Supplementary Materials The aim of the Supplementary Materials is to introduce SAS codes for multilevel modeling on the association between local statistics of physical activity based on accelerometer data and self-reported affect based on EMA. It consists of three parts: (a) Aggregating acceleration counts to various time windows (e.g., 60 min). (b) Merging physical activity data with self-reported affect. (c) Reporting the code for multilevel modeling on the association between local statistics of physical activity and affect, as described in Section 2.1.
