# Supplementary material for: A Practical Guide to Analyzing Time-Varying Associations between Physical Activity and Affect Using Multilevel Modeling
Source: Comput Math Methods Med. 2018 Jul 9;2018:8652034. doi: 10.1155/2018/8652034 (PMC6076963; doi:10.1155/2018/8652034)
Supplement: Supplementary Materials — The aim of the Supplementary Materials is to introduce SAS codes for multilevel modeling on the association between local statistics of physical activity based on accelerometer data and self-reported affect based on EMA. It consists of three parts: (a) Aggregating acceleration counts to various time windows (e.g., 60 min). (b) Merging physical activity data with self-reported affect. (c) Reporting the code for multilevel modeling on the association between local statistics of physical activity and affect, as described in Section 2.1. [file 8652034.f1.docx]

Supplementary Materials

The aim of the Supplementary Materials is to introduce SAS codes for multilevel modeling on the association between local statistics of physical activity (PA) based on accelerometer data and self-reported affect based on ecological momentary assessment (EMA). It consists of three parts:

1. Aggregating acceleration counts to various time windows (e.g., 60 min).
2. Merging PA data with self-reported affect.
3. Reporting the code for multilevel modeling on the association between local statistics of PA and affect, as described in section 2.1.

*Key variables we used in the SAS codes

- Epoch: Time information (minute) from the beginning to the end of data collection
- TotAC: Total activity counts within a certain time window (e.g., 60-min time window)
- ACsed: **Activity counts for sedentary behaviors in a certain time window**
- AClig: **Activity counts for light intensity PA in a certain time window**
- ACmod: **Activity counts for moderate intensity PA in a certain time window**
- ACvig: **Activity counts for vigorous intensity PA in a certain time window**
- TotEE: Total energy expenditure within a certain time window
- EEsed: **Energy expenditure for sedentary behaviors in a certain time window**
- EElig: **Energy expenditure for light intensity PA in a certain time window**
- EEmod: **Energy expenditure for moderate intensity PA in a certain time window**
- EEvig: **Energy expenditure for vigorous intensity PA in a certain time window**

/****************************************************************************

* a. Aggregating acceleration counts to various time windows *

* (e.g., 60 min) *

****************************************************************************/

*Specifying path to the data file and transferring it from .xlsx to .sas7bdat;

**proc** **import** out=raw_SAS_data

datafile="C:\Users\Jinhyuk Kim\CMMM\datasets\raw_EXCEL_data.xlsx"

dbms=xlsx;

sheet="data";

**run**;

*Checking raw activity data for each ID;

**proc** **template**;

define statgraph plot;

begingraph;

layout overlay;

barchartparm x=time_min y=activity;

endlayout;

endgraph;

end;

**run**;

**proc** **sgrender** data=raw_SAS_data template=plot;

by id;

**run**;

*Cleaning up PA data where subjects took off the device (or where false assessments detected);

*Because we used the device that can measure PA and heart rate at the same time, if there is no valid heart rate data, we considered it as taking off time or false assessments. It should be considered how to address invalid activity data according to the device used in your study;

**data** cleaned_act; set raw_SAS_data;

if Heart_Rate='NaN' then do;

TotAC=**.**; TotEE=**.**;

end;

**run**;

*Generating shared time information throughout the study for merging datasets;

**data** epoch_info; set cleaned_act;

id_epoch = id + epoch_***0.000001**;

**run**;

*TotAC and TotEE are divided into four different intensity levels of PA based on cut-off points. Original cut-off points provided by the device maker were used in this code, but cut-off points validated by prior studies should be recommended;

**data** cutoff_act; set epoch_info;

*Classification based on the intensity of activity counts;

if CutOff=**1** then ACsed=TotAC; else ACsed=**.**;

if CutOff=**2** then AClig=TotAC; else AClig=**.**;

if CutOff=**3** then ACmod=TotAC; else ACmod=**.**;

if CutOff=**4** then ACvig=TotAC; else ACvig=**.**;

*Classification based on the intensity of energy expenditure;

if CutOff=**1** then EEsed=TotEE; else EEsed=**.**;

if CutOff=**2** then EElig=TotEE; else EElig=**.**;

if CutOff=**3** then EEmod=TotEE; else EEmod=**.**;

if CutOff=**4** then EEvig=TotEE; else EEvig=**.**;

**run**;

*Specifying path for saving temporary and final activity data files;

libname save "C:\Users\Jinhyuk Kim\CMMM\datasets";

*Aggregation for local statistics of PA before the time EMA assessed (e.g., when aggregating 60-min duration of PA before 10:00, the time window will be from 9:00 [-60 min] to 9:59 [-1 min]). Activity counts and energy expenditure were accumulated every 1-min epoch in the example data set;

**%macro** activity_before(start,interval,end);

%do i=&start. %to &end. %by &interval.;

*Start: start point(min) for aggregating activity counts (e.g., if start=10, aggregation starts with a time window from -1 to -10 min before the time EMA assessed);

*Interval: increase of window size(min) for aggregating activity counts (e.g., if interval=10, the window size of aggregation increases by 10 min [i.e., 10,20,30...]);

*End: endpoint(min) for aggregating activity counts (e.g., if end=120, aggregation is made up to the window size of 120 min);

proc sql;

create table tmp_act_B_&i. as

select l.*

, sum(r.TotAC) as S_TotAC_B_&i.

, count(r.TotAC) as N_TotAC_B_&i.

, count(r.ACsed) as N_ACsed_B_&i.

, mean(r.TotAC) as M_TotAC_B_&i.

, max(r.TotAC) as P_TotAC_B_&i.

from cutoff_act L left join cutoff_act R

on l.ID=r.ID and r.Epoch >= l.Epoch-&i. and r.Epoch < l.Epoch

group by l.ID, l.Epoch, l.TotAC, l.ACsed;

quit;

data save.act_B_%eval(&i./&interval.); set tmp_act_B_&i.;

by ID_epoch;

if not first.ID_epoch then delete;

run;

%end;

*Merging all aggregations from the macro function;

data act_merge_A;

merge save.act_A_1-save.act_A_%eval(&end./&interval.);

by id_epoch;

run;

**%mend**;

%***activity_before***(start=**10**,interval=**10**,end=**120**) *Aggregation is made using time windows from 10 min to 120 min with a 10-min interval before the time EMA assessed;

*Aggregation for local statistics of PA after the time EMA assessed;

**%macro** activity_after (start,interval,end);

%do i=&start. %to &end. %by &interval.;

proc sql;

create table temp_act_A_&i. as

select l.*

, sum(r.TotAC) as S_TotAC_A_&i.

, count(r.TotAC) as N_TotAC_A_&i.

, count(r.ACsed) as N_ACsed_A_&i.

, mean(r.TotAC) as M_TotAC_A_&i.

, max(r.TotAC) as P_TotAC_A_&i.

from cutoff_act L left join cutoff_act R

on l.ID=r.ID and r.Epoch <= l.Epoch+&i. and r.Epoch > l.Epoch

group by l.ID, l.Epoch, l.TotAC, l.ACsed;

quit;

data save.act_A_%eval(&i./&interval.); set temp_act_A_&i.;

by ID_epoch;

if not first.ID_epoch then delete;

run;

%end;

data act_merge_B;

merge save.act_A_1-save.act_B_%eval(&end./&interval.);

by id_epoch;

run;

**%mend**;

%***activity_after***(start=**5**,interval=**5**,end=**120**) *Aggregation is made using time windows from 5 min to 120 min with a 5-min interval after the time EMA assessed;

*Sorting the data sets you want to merge;

**proc** **sort** data=act_merge_B out=sorted_act_B;

by id_epoch;

**run**;

**proc** **sort** data=act_merge_A out=sorted_act_A;

by id_epoch;

**run**;

*Generating one PA data before/after the time EMA;

**data** act_data;

merge sorted_act_B sorted_act_A;

by id_epoch;

**run**;

*The local statistics of PA around the time EMA assessed can be aggregated later with the local statistics after/before the time EMA assessed (e.g., 60-min local mean around EMA = [30-min local mean before EMA + 30-min local mean after EMA]/2);

**data** act_around; set act_data;

M_TotAC_AR_60 = (M_TotAC_B_30 + M_TotAC_A_30)/**2**;

**run**;

/****************************************************************************

* b. Merging PA data with affect *

****************************************************************************/

*Specifying path to EMA data;

libname dataset "C:\Users\Jinhyuk Kim\CMMM\datasets";

*Generating shared time information to merge with PA data;

**data** EMA_data; set dataset.raw_EMA_data;

h = hour(ematime);

m = minute(ematime);

ema_min = h***60** + m;

epoch = studyday***1440** + ema_min; *1440=24h*60min;

id_epoch = id + epoch***0.000001**;

**run**;

*Sorting the data sets you want to merge;

**proc** **sort** data=EMA_data out=sorted_ema;

by id_epoch;

**run**;

**proc** **sort** data=act_prop out=sorted_act;

by id_epoch;

**run**;

*Merging PA data with EMA;

**data** ema_act_merge;

merge sorted_ema sorted_act;

by id_epoch;

**run**;

/****************************************************************************

* c. Reporting the code for analyzing the association between local *

* statistics of PA and affect, as described in section 2.1 *

****************************************************************************/

* Generating a PDF file for SAS results;

ODS pdf file="C:\Users\Jinhyuk Kim\CMMM\results";

%let DV = M_TotAC_B_60 M_TotAC_AR_60 M_TotAC_A_60 M_TotEE_AR_60 M_TotEE_A_60;

%let Predictor = NA Depression; * NA: Negative Affect;

**%macro** ***MLM***;

%do i=**1** %to %eval(%sysfunc(countc(&DV., " "))+**1**);

%let DV1 = %scan(&DV, &i);

%put DV1;

%do j=**1** %to %eval(%sysfunc(countc(&predictor., " "))+**1**);

%let predictor1 = %scan(&predictor, &j);

%put predictor1;

*Generating person mean of predictors;

proc means data=ema_act_merge nway noprint;

by ID;

var NA;

output out=person_mean

mean(&predictor1.)=&predictor1._pm;

run;

data merged_pm;

merge ema_act_merge person_mean;

by ID;

run;

*Generating person-mean centered predictors;

data final_data; set merged_pm;

&predictor1._pmc = &predictor1. - &predictor1._pm;

run;

*2-level multilevel modeling;

title &DV1. -> &predictor1.;

proc mixed data=pa_analysis method=ml noclprint noitprint covtest;

class id;

model &DV1. = &predictor1. / solution ddfm=bw;

random intercept &predictor1./subject=id type=un gcorr;

run;

*3-level multilevel modeling;

title &DV1. -> &predictor1.;

proc mixed data=pa_analysis method=ml noclprint noitprint covtest;

class id;

model &DV1. = &predictor1. &predictor1._pm / solution ddfm=bw;

random intercept &predictor1./subject=id type=un gcorr;

random intercept &predictor1./subject=Studyday(id);

run;

%end;

%end;

**%mend**;

%***MLM***;

ods pdf close;
